# Estimation of the number of exposed people during highly pathogenic avian influenza virus outbreaks in EU/EEA countries, October 2016–September 2018

**DOI:** 10.1111/zph.12629

**Published:** 2019-09-06

**Authors:** Cornelia Adlhoch, Aleksandra Miteva, Anna Zdravkova, Tihana Miškić, Dražen Kneževic, Sokratis Perdikaris, Krzysztof Śmietanka, Edyta Świętoń, Vilem Kopriva, Martin Chudý, Luis Jose Romero González, Ines Moreno Gil, Annica Wallén Norell, Frank Verdonck

**Affiliations:** ^1^ European Centre for Disease Prevention and Control Solna Sweden; ^2^ Bulgarian Food Safety Agency Sofia Bulgaria; ^3^ Ministry of Agriculture Veterinary and Food Safety Directorate Zagreb Croatia; ^4^ Croatian Agency for Agriculture and Food Osijek Croatia; ^5^ Ministry of Rural Development and Food, General Directorate of Veterinary Services Directorate of Animal Health Athens Greece; ^6^ National Veterinary Research Institute Puławy Poland; ^7^ State Veterinary and Food Administration of the Slovak Republic Bratislava Slovakia; ^8^ Ministry of Agriculture Fisheries and Food Madrid Spain; ^9^ Jordbruksverket Jönköping Sweden; ^10^ European Food Safety Authority Parma Italy

**Keywords:** A(H5N8), avian influenza, EU/EEA, human exposure

## Abstract

We estimated that more than 11,000 people were exposed to highly pathogenic avian influenza viruses in EU/EEA countries over the outbreak period October 2016–September 2018 by cross‐linking data submitted by Member States to European Food Safety Authority and EMPRES‐i. A stronger framework for collecting human exposure data is required.

**Impact**

- Estimation of the magnitude of human exposure during avian influenza outbreaks October 2016–September 2018 results in more than 11,000 people being involved.
- Majority of human exposures were related to outbreaks in commercial holdings with more than 10,000 birds.
- Level of protection might be higher for people exposed during outbreaks in holdings with large number of poultry than for people exposed to infected single birds, which for the latter might not be as easily identified or followed up by public health authorities.

## 1 INTRODUCTION

Between 2016 and 2018, outbreaks of highly pathogenic avian influenza virus (HPAI) A(H5N8), A(H5N6) and A(H5N5) affected 24 EU/EEA countries (EFSA, Brown, Kuiken, et al. 2017; EFSA, Brown, Mulatti, et al., 2017). Approximately 20 million birds had to be culled either directly due to a confirmed outbreak or indirectly as a precaution, for example, related to prevention measures within the control zone (Hansen, Brown, Brookes, Welchman, & Cromie, 2018; Napp, Majo, Sanchez‐Gonzalez, & Vergara‐Alert, 2018). Emerging avian influenza viruses pose a risk of transmission to humans, particularly through unprotected direct contact between humans and infected birds (Tate, 2018). Information of significant human exposure and early detection of bird‐to‐human transmission events is crucial to prevent potential onward spread in the community. It is also necessary to link information between avian influenza outbreaks and human exposure for better evidence generation and public health risk assessment. A previous study did not identify any transmission to people exposed during the initial phase of the outbreaks (Adlhoch, Dabrera, Penttinen, Pebody, & Country, 2018), and until now, no human infection related to these avian influenza viruses of clade 2.3.4.4 has been reported in Europe. The Food and Agriculture Organization of the United Nations (FAO) made data on all avian influenza virus outbreaks publicly available through the Global Animal Disease Information System (EMPRES‐i) (2018). As the total number of people exposed during the outbreaks is not available, we attempt to extrapolate the number of people exposed to HPAI viruses A(H5N5), A(H5N6) and A(H5N8) in EU/EEA Member States over the period 1 October 2016–26 September 2018 by cross‐linking different data sets.

## 2 THE STUDY

The European Food Safety Authority (EFSA) asked all EU/EEA countries’ representatives to provide data on HPAI outbreaks for the period October 2016–June 2017 (Brown, Mulatti, et al. (2017)). Reporting categories included the number of susceptible birds, holding specifications (commercial, non‐commercial) and the number of people exposed. When multiple records with the same unique identifier of a holding were entered in the data set, for example when holdings with several different bird species were affected, the numbers of birds were summed up and the mean number of exposed people per unique identifier was used. Entries with missing number of susceptible birds or humans exposed were excluded. We categorized the number of susceptible birds (0–50, 51–200, 201–1,000, 1,001–10,000 and >10,000). The sum and mean number of exposed people were calculated for each category.

During the respective period, 19 countries reported outbreaks or wild bird findings to EFSA, and of those, nine countries provided information on human exposure. Overall 1,293 people were reported to be exposed during 200 events, 960 during 100 events in commercial holdings and 333 during 100 events in non‐commercial settings (Table 1). All but one event, where A(H5N6) was reported, were due to A(H5N8). The vast majority of events in commercial holdings affected farms with more than 1,000 birds and therefore required nearly three times more people than events in non‐commercial settings which mostly affected holdings with up to 200 birds. Single wild bird findings contributed to 61% of all events significantly to the overall exposure. A higher number of people with exposure were reported in events affecting higher number of animals, which indicates that culling activities in large settings require the involvement of more people to manage the situation, although the numbers per event in each size category differ between the countries. This relates to the characteristics of the respective poultry production industry in the countries and to the different management practices in such settings related to HPAI viruses.

**Table 1:** Number of highly pathogenic avian influenza A(H5N8), A(H5N6) and A(H5N5) virus events in animals and people exposed by number of susceptible animals and commercial or non‐commercial nature of establishments, EU/EEA, 1 October 2016–26 September 2018

Susceptible animals	EFSA data						EMPRES‐i data		Extrapolated number of people exposed		
	Commercial			Non‐commercial			Number of outbreaks		Commercial^c^ (95%CI)	Non‐commercial^d^ (95%CI)	Total (95%CI)
	Number of outbreaks	Sum of people exposed	Mean number/outbreak (95%CI)	Number of outbreaks	Sum of people exposed	Mean number/outbreak (95%CI)	Commercial^a^	Non‐commercial^b^			
1–50	1	3	3.0	49	139	2.8 (2.5–3.1)	342	955	1,026	2,674 (2,422–2,996)	3,700 (2,422–2,996)
51–200	3	14	4.7 (−2.9 to 12.3)^e^	37	141	3.8 (3.2–4.4)	95	13	443 (0–1,164)	50 (42–57)	493 (42–1,221)
201–1,000	7	52	7.4 (3.2 to 11.7)	13	49	3.8 (2.8–4.8)	108	3	802 (341–1,263)	11 (8–14)	814 (350–1,277)
1,001–10,000	46	264	5.7 (3.9 to 7.6)	1	4	4	344	0	1,974 (1,349–2,599)		1,974 (1,349–2,599)
>10,000	43	627	14.6 (10.1 to 19.1)	0	0	—	282	0	4,112 (2,851–5,373)		4,112 (2,851–5,373)
Total	100	960	—	100	333	—	1,171	971	8,358 (4,542–10,400)	2,735 (2,472–3,068)	11,093 (7,014–13,468)

Note

EFSA data set comprised only data from nine MS (October 2016–June 2017), EMPRES‐i data: 01/10/2016 and 26/09/2018.

Abbreviation: EFSA, European Food Safety Authority; 95% CI, 95% confidence interval.

a Domestic birds.

b Captive and wild birds.

c Mean number of people exposed in commercial establishments (EFSA) multiplied by number of events in commercial establishments (EMPRES‐i).

d Mean number of people exposed in non‐commercial establishments (EFSA) multiplied by number of events in non‐commercial establishments (EMPRES‐i).

e Calculated as 0 in the table for the 95% CI estimates.

Data available on events due to HPAI viruses A(H5N5), A(H5N6) and A(H5N8) between 01/10/2016 and 26/09/2018 in EU/EEA countries were downloaded from the EMPRES‐i database (26 September 2018). Entries that listed a bird species but missed the number of affected birds were considered a single event. We analysed the number of events by bird classification (captive, domestic, wild), using the same categories for the number of affected animals as described above.

**Figure 1:**
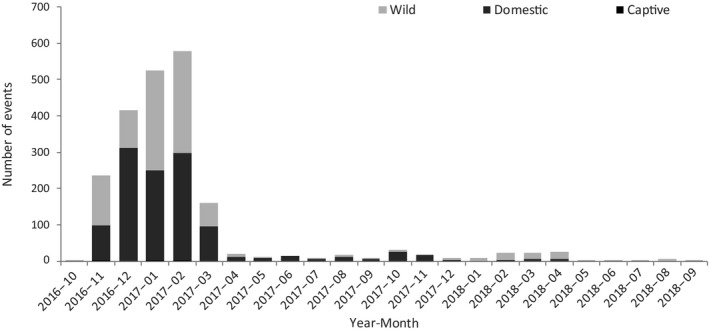
Number of highly pathogenic avian influenza A(H5N8), A(H5N6) and A(H5N5) virus events in birds, by outbreak type and month‐year of the outbreak detection, EU/EEA, 1 October 2016–26 September 2018

The EMPRES‐i database listed a total of 2,142 events involving 10,933,763 affected birds for the period 1 October 2016–26 September 2018 in 24 EU/EEA countries (Table 1). The majority (1,171; 55%) of these events were related to domestic birds, 24% (282) of the latter occurred in bird populations >10,000 animals. Detections in wild birds contributed substantially to the number of events overall (962; 45%), with 99% (951) in single birds to small groups of birds (1–50 animals). Only a few events (9) were reported in captive birds (Figure 1).

For the extrapolation of the total number of people who were exposed, we multiplied the mean number of people exposed per event from the EFSA data set with the reported total number of events in each size category from the EMPRES‐i data and by commercial or non‐commercial nature of the establishment. This resulted in an estimate of 11,093 (95% CI: 7,014–13,468) people exposed during the events (Table 1). If the calculation is performed without any stratification by multiplying the mean number of exposed with the total number of outbreaks, a slightly higher estimate results with 13,848 (95% CI: 11,273–16,422) people being exposed. The vast majority (75%) of human exposures were related to events in commercial holdings, and more than 50% of the latter occurred at holdings with more than 10,000 birds.

## 3 DISCUSSION

According to our estimate, more than 11,000 people were exposed to infected birds during the avian influenza outbreaks between October 2016 and September 2018 in EU/EEA countries. It is not surprising that control activities in large holdings require extensively more human resources than those in small holdings or single events and account for the highest number of humans exposed. However, 33% of the exposure were related to single bird (and up to 50) detections. On the one hand, people involved in culling activities in large establishments are much more intensively exposed to large numbers of infected birds for longer periods in enclosed facilities than people who respond to a dead wild bird. On the other hand, the level of personal protection (including face shields, full body cover and gloves) during such ordered culling activities in specialized establishments might be higher than for sporadic detections in nature or in backyards. People exposed to single birds might also not be as easily identified or followed up by public health authorities.

The extrapolated estimate of around 11,000 people is unadjusted, and several factors of uncertainty or missing information limit this analysis. This includes the accuracy of the reporting of HPAI in wild birds as well as the completeness of the estimated number of exposed persons and affected birds. The different approaches in the country regarding organization of culling activities and outbreak management practice might also bias the estimation as previously described (Adlhoch et al., 2016; Adlhoch, Dabrera, et al., 2018). It was not known whether the same people (specialized culler teams) were involved in many different culling operations and were thus counted multiple times leading to an overestimation of the total number. However, each involvement in an operation would represent an exposure situation providing an occasion for virus transmission. Also, no detailed information on the duration of the exposure during culling activities nor on personal protection measures was available that would have allowed to rank the level of exposure. As not all outbreaks may have been reported, our figures might rather underestimate the true magnitude.

During the outbreak period, a higher number of wild birds were affected over a longer period compared to affected commercial establishments during previous outbreaks of avian influenza in Europe (Alkhamis, Moore, & Perez, 2015; Walsh, Amstislavski, Greene, & Haseeb, 2017). Whereas traditional education has focused mainly on large poultry facilities, our findings suggest more outreach to other groups is needed, such as hunters or persons exposed to wild birds. The data provided might also be useful for the countries to better prepare in terms of staff and resources needed for a response in the future particularly when large HPAI outbreaks occur during seasonal influenza epidemics. Although the currently detected avian influenza viruses might have a low risk of transmission (EFSA et al., 2018; Grund et al., 2018), it cannot be excluded that viruses with higher zoonotic potential might be introduced into Europe in the future. In such a case, it is necessary to identify those at risk for early intervention and control measures as well as to collect detailed information on the duration and level of exposure as well as protection measures for better evidence generation. Collecting the number of exposed people remains challenging due to split responsibilities between relevant authorities in the different sectors at local, regional and national levels. Stronger frameworks for collecting and sharing comprehensive human exposure data, in particular between the animal and human health sectors, for early response are therefore crucial.

## 4 DISCLAIMER

The manuscript has been developed and published under the sole responsibility of the authors. It must not be considered as an EFSA scientific output and does not necessarily represent any official position of EFSA.

## CONFLICT OF INTEREST

None to declare.

## References

[zph12629-bib-0002] Adlhoch, C. , Brown, I. H. , Angelova, S. G. , Bálint, Á. , Bouwstra, R. , Buda, S. , … Penttinen, P. (2016). Highly pathogenic avian influenza A(H5N8) outbreaks: Protection and management of exposed people in Europe, 2014/15 and 2016. Eurosurveillance, 21(49). 10.2807/1560-7917.ES.2016.21.49.30419 PMC5291128 27983512

[zph12629-bib-0003] Adlhoch, C. , Dabrera, G. , Penttinen, P. , Pebody, R. , & Country, E. (2018). Protective measures for humans against avian influenza A(H5N8) outbreaks in 22 European Union/European Economic Area Countries and Israel, 2016–17. Emerging Infectious Diseases, 24, 1–8. 10.3201/eid2410.180269 PMC6154149 29989531

[zph12629-bib-0004] Alkhamis, M. A. , Moore, B. R. , & Perez, A. M. (2015). Phylodynamics of H5N1 highly pathogenic avian influenza in Europe, 2005–2010: Potential for molecular surveillance of new outbreaks. Viruses, 7, 3310–3328. 10.3390/v7062773 26110587 PMC4488740

[zph12629-bib-0001] EFSA (European Food Safety Authority) , ECDC (European Centre for Disease Prevention and Control) , EURL (European Reference Laboratory for Avian Influenza) , Adlhoch, C. , Brouwer, A. , Kuiken, T. , … Baldinelli, F. (2018). Scientific report: Avian influenza overview November 2017 – February 2018. EFSA Journal, 16, e05240 10.2903/j.efsa.2018.5240 PMC7009675 32625858

[zph12629-bib-0005] EFSA (European Food Safety Authority) , ECDC (European Centre for Disease Prevention and Control) , EURL (European Reference Laboratory on Avian Influenza) , Brown, I. , Kuiken, T. , Mulatti, P. , … Adlhoch, C. (2017). Scientific report: Avian influenza overview September ‐ November 2017. EFSA Journal, 15, e05141 10.2903/j.efsa.2017.5141 PMC7010192 32625395

[zph12629-bib-0006] EFSA (European Food Safety Authority) , ECDC (European Centre for Disease Prevention and Control) , EURL (European Union Reference Laboratory for Avian influenza) , Brown, I. , Mulatti, P. , Smietanka, K. , … Verdonck, F. (2017). Scientific report on the avian influenza overview October 2016–August 2017. EFSA Journal, 15, e05018. 10.2903/j.efsa.2017.5018 PMC7009863 32625308

[zph12629-bib-0007] Food and Agriculture Organization of the United Nations (FAO) . (2018). Global Animal Disease Information System (EMPRES‐i), Rome, Italy. Retrived from http://empres-i.fao.org/eipws3g/

[zph12629-bib-0008] Grund, C. , Hoffmann, D. , Ulrich, R. , Naguib, M. , Schinkothe, J. , Hoffmann, B. , … Beer, M. (2018). A novel European H5N8 influenza A virus has increased virulence in ducks but low zoonotic potential. Emerging Microbes and Infections, 7, 132 10.1038/s41426-018-0130-1 30026505 PMC6053424

[zph12629-bib-0009] Hansen, R. , Brown, I. , Brookes, S. , Welchman, D. , & Cromie, R. (2018). Current status of avian influenza in Europe and the UK. The Veterinary Record, 182, 54–55. 10.1136/vr.k128 29326393

[zph12629-bib-0010] Napp, S. , Majo, N. , Sanchez‐Gonzalez, R. , & Vergara‐Alert, J. (2018). Emergence and spread of highly pathogenic avian influenza A(H5N8) in Europe in 2016–2017. Transboundary and Emerging Diseases, 65(5), 1217–1226. 10.1111/tbed.12861 29536643

[zph12629-bib-0011] Tate, M. D. (2018). Highly pathogenic avian H5N8 influenza viruses: Should we be concerned? Virulence, 9, 20–21. 10.1080/21505594.2017.1386832 28968185 PMC5801643

[zph12629-bib-0012] Walsh, M. G. , Amstislavski, P. , Greene, A. , & Haseeb, M. A. (2017). The Landscape epidemiology of seasonal clustering of highly pathogenic avian influenza (H5N1) in domestic poultry in Africa, Europe and Asia. Transbound Emerg Dis, 64, 1465–1478. 10.1111/tbed.12537 27311569

